# Tumor-Agnostic Circulating Tumor DNA Testing for Monitoring Muscle-Invasive Bladder Cancer

**DOI:** 10.3390/ijms242316578

**Published:** 2023-11-21

**Authors:** Raquel Carrasco, Mercedes Ingelmo-Torres, Ramón Trullas, Fiorella L. Roldán, Leonardo Rodríguez-Carunchio, Lourdes Juez, Joan Sureda, Antonio Alcaraz, Lourdes Mengual, Laura Izquierdo

**Affiliations:** 1 Laboratori i Servei d’Urologia, Hospital Clinic de Barcelona, 08036 Barcelona, Spain; racarrasco@recerca.clinic.cat (R.C.); ingelmo@recerca.clinic.cat (M.I.-T.); flroldan@clinic.cat (F.L.R.); ljuez@clinic.cat (L.J.); josureda@clinic.cat (J.S.); aalcaraz@clinic.cat (A.A.); lizquier@clinic.cat (L.I.); 2 Genètica i Tumors Urològics, Fundació de Recerca Clinic Barcelona-Institut d’Investigacions Biomèdiques August Pi i Sunyer (FRCB-IDIBAPS), 08036 Barcelona, Spain; 3 Departament de Biomedicina, Facultat de Medicina i Ciències de la Salut, Universitat de Barcelona (UB), 08036 Barcelona, Spain; 4 Unitat de Neurobiologia, Institut d’Investigacions Biomèdiques de Barcelona (IIBB/CSIC/IDIBAPS), 08036 Barcelona, Spain; ramon.trullas@iibb.csic.es; 5 Servei d’Anatomia Patològica, Hospital Clinic de Barcelona, 08036 Barcelona, Spain; lerodrig@clinic.cat

**Keywords:** bladder cancer, circulating tumor DNA, droplet digital PCR, prognosis

## Abstract

Circulating tumor DNA (ctDNA) has recently emerged as a real-time prognostic and predictive biomarker for monitoring cancer patients. Here, we aimed to ascertain whether tumor-agnostic ctDNA testing would be a feasible strategy to monitor disease progression and therapeutic response in muscle-invasive bladder cancer (MIBC) patients after radical cystectomy (RC). Forty-two MIBC patients who underwent RC were prospectively included. Blood samples from these patients were collected at different follow-up time points. Two specific mutations (*TERT* c.1-124C>T and *ATM* c.1236-2A>T) were analyzed in the patients’ plasma samples by droplet digital PCR to determine their ctDNA status. During a median follow-up of 21 months, 24% of patients progressed in a median of six months. ctDNA status was identified as a prognostic biomarker of tumor progression before RC and 4 and 12 months later (HR 6.774, HR 3.673, and HR 30.865, respectively; *p* < 0.05). Lastly, dynamic changes in ctDNA status between baseline and four months later were significantly associated with patient outcomes (*p* = 0.045). In conclusion, longitudinal ctDNA analysis using a tumor-agnostic approach is a potential tool for monitoring MIBC patients after RC. The implementation of this testing in a clinical setting could improve disease management and patients’ outcomes.

## 1. Introduction

Muscle-invasive bladder cancer (MIBC) is a heterogeneous and aggressive disease with a poor prognosis. Neoadjuvant chemotherapy (for tumors that are cisplatin-eligible) coupled with radical cystectomy (RC) and lymphadenectomy is the gold standard treatment for localized MIBC [1]. Specific patients might receive adjuvant therapies to reduce tumor progression [1]. Despite these treatments, approximately 50% of MIBC patients will develop local or distant metastases within two years after RC [1,2]. Currently, treatment response monitoring, as well as the detection of metastatic relapse, is performed via imaging techniques [1]. However, imaging background and lesion size could limit the detection of tumor dissemination [3]. Therefore, the identification of prognostic and predictive biomarkers that help detect metastatic relapse earlier and guide treatment would directly impact MIBC patient outcomes.

Circulating tumor DNA (ctDNA) has recently emerged as a real-time prognostic and predictive biomarker for monitoring cancer patients with different tumor types [4,5,6,7,8], including BC [9,10,11,12]. Currently, the detection of ctDNA can be based on two approaches: the tumor-informed approach, which requires the initial genomic profiling of the primary tumor, and the tumor-agnostic approach, which is independent of the genomic knowledge of the patient’s prior tumor. Previously, we evaluated the potential prognostic utility of a tumor-informed ctDNA approach to monitor MIBC patients after RC [13]. For this purpose, we analyzed four specific mutations, previously identified in tumor tissue [14], in plasma samples from 37 MIBC patients at different follow-up time points after RC. We found that ctDNA status four months after RC is a prognostic biomarker of patients’ clinical outcomes. Moreover, we showed that dynamic ctDNA status can detect tumor progression earlier than imaging techniques [13]. Therefore, we demonstrated that ctDNA status could be a useful tool for monitoring MIBC patients after RC.

Here, we aimed to ascertain whether tumor-agnostic ctDNA testing would be a feasible strategy to monitor tumor progression and therapeutic response in MIBC patients after RC. The great advantage of this approach is that it would allow monitoring of MIBC patients, avoiding individual tumor analysis and patient-specific ctDNA assays, thus shortening and simplifying the methodological protocols and promoting the transfer of the approach to clinical practice.

## 2. Results

### 2.1. Clinicopathological Features of the Series

The clinicopathological characteristics of the MIBC patients enrolled in this study are shown in Table 1. During a median follow-up of 21 months (range 6–37 months), 10 (24%) patients progressed (seven with LN+); the median time to progression was six months (range 1–11 months). Three of the ten progressive patients received neoadjuvant chemotherapy, and a fourth received adjuvant chemotherapy; the six remaining progressive patients were unfit for chemotherapy. Overall, 50% (5/10) of progressive patients received treatment during tumor progression. During follow-up, eight (19%) patients died, six (75%) due to the BC. The median time of cancer-specific survival (CSS) was 12 months (range 6–21 months).

### 2.2. Analysis of TERT and ATM Mutations in cfDNA

The percentage of patients with *TERT* or *ATM* mutations identified in cfDNA samples according to each follow-up time point is depicted in Figure S1. As shown, there was a higher percentage of patients with *TERT* mutations at all follow-up time points. The mean mutant allele fraction (MAF) of the *TERT* mutation was significantly higher in progressive than in non-progressive MIBC patients at baseline and 12 months later (Figure S2A). In addition, the mean MAF of the *TERT* mutation was also significantly higher in those patients who died due to the BC at baseline and 12 months later (Figure S2B). No significant differences were found for the mean MAF of the *ATM* mutation according to tumor progression or death occurrence at any follow-up time point (Table S1).

### 2.3. Prognostic Value of ctDNA Status

**Figure 1:**
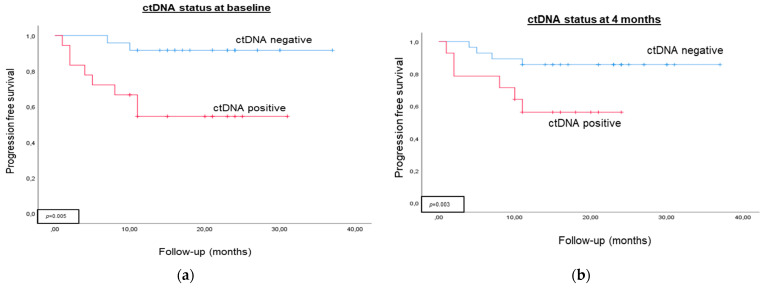

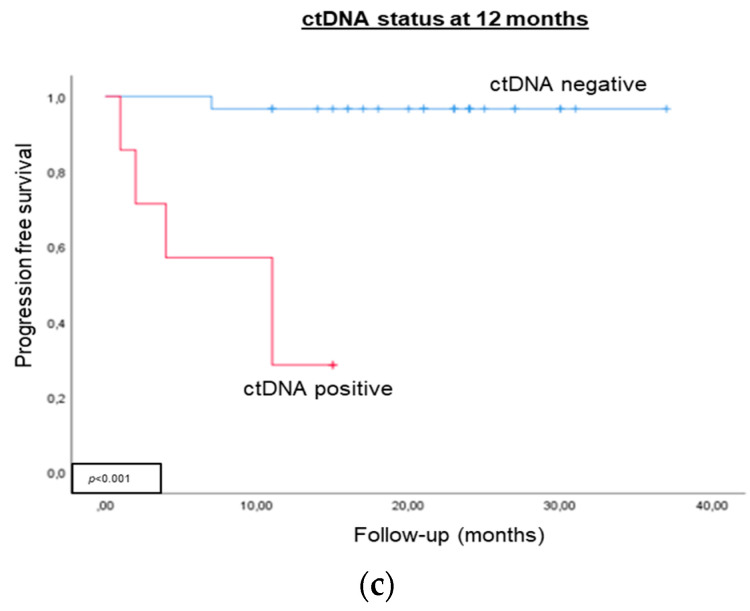
Prognostic value of ctDNA status at different time points. The Kaplan–Meier survival analysis shows the probability of tumor progression in MIBC patients stratified by ctDNA status (**a**) at baseline, (**b**) four months after RC, and (**c**) 12 months after RC.

Cox regression analysis evaluated ctDNA status at each follow-up time point and clinicopathological variable. Univariate Cox regression analysis identified positive ctDNA status as a prognostic biomarker of tumor progression before RC and 4 and 12 months later (HR 6.774, HR 3.673, and HR 30.865, respectively; *p* < 0.05). Figure 1 depicts Kaplan–Meier curves showing that ctDNA status can discriminate between two groups of MIBC patients with a significantly different probability of tumor progression before RC and during patient follow-up. Contrarily, ctDNA status was not found to be a prognostic biomarker of CSS at any follow-up time point.

Overall, the subgroup of MIBC patients harboring the *TERT* c.1-124C>T mutation had a worse clinical outcome than those without the mutation (Figure S3). Specifically, MIBC patients harboring this *TERT* mutation before RC and/or four months later were at higher risk of tumor progression than those patients without this mutation at these follow-up time points (HR 5.761, *p* = 0.012). Additionally, MIBC patients harboring the *TERT* c.1-124C>T mutation at both follow-up time points had the highest risk of tumor progression compared with the other MIBC patients (HR 6.405, *p* = 0.002).

### 2.4. Prognostic Value of Dynamic Changes in ctDNA Status

**Figure 2:**
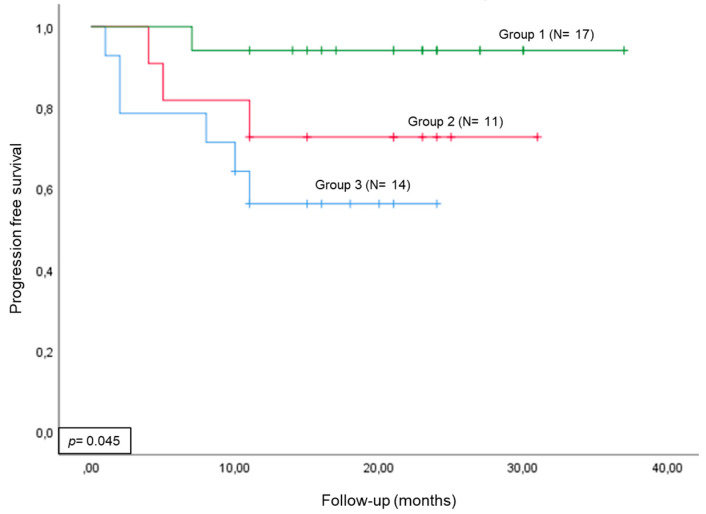
Dynamic ctDNA status analysis. Kaplan–Meier curve for tumor progression in three different MIBC groups stratified by dynamic changes in ctDNA status between baseline and four months after RC: Group 1, patients with negative ctDNA status at baseline and later; Group 2, patients with positive ctDNA status at baseline and negative ctDNA status four months after RC (ctDNA clearance); and Group 3, patients with positive ctDNA status at four months.

The patients were stratified into three groups according to their ctDNA status before RC and four months later: Group (1) patients with negative ctDNA status at baseline and at four months; Group (2) patients with positive ctDNA status at baseline and negative ctDNA status four months after RC (ctDNA clearance); and Group (3) patients with positive ctDNA status at four months. As shown in Figure 2, dynamic changes in ctDNA status were significantly associated with the patients’ clinical outcomes. In brief, those patients with a positive ctDNA status four months after RC (Group 3) had the poorest clinical outcome. On the other hand, those patients with ctDNA clearance after RC (Group 2) had significantly longer progression-free survival (PFS) than those from Group 3. Finally, patients with undetectable ctDNA in their bloodstream (Group 1) had the best clinical outcome.

## 3. Discussion

Recently, ctDNA has become a powerful biomarker in oncology because of its utility in monitoring molecular relapse and treatment response or detecting molecular residual disease in multiple cancer types [4,8,15,16], including BC [9,12,17,18,19,20,21]. Using a tumor-informed approach to evaluate serial cfDNA samples from MIBC patients [13], we were able to demonstrate that a positive ctDNA status four months after RC had prognostic implications. We also showed that ctDNA clearance after surgery was significantly associated with patients’ clinical outcomes. Moreover, we identified that ctDNA status could predict metastatic relapse earlier than imaging techniques. Therefore, we concluded that ctDNA status could be a potential prognostic biomarker for monitoring MIBC patients after RC. However, this prior study collectively relied on a tumor-informed approach, challenging the translation of these personalized methodological tests to clinical settings because of their evidently high economic cost and workload. The development of a ctDNA assay composed of a panel of the most frequently mutated BC genes in these tumors would avoid assessing patient-specific variants, thus simplifying the methodological process with a single ctDNA assay to monitor all patients.

To the best of our knowledge, the present work is the first to evaluate the prognostic utility of tumor-agnostic ctDNA testing for monitoring MIBC patients after RC using a reduced gene panel targeting a few BC-specific genes. Previous works from other authors on solid tumors such as breast [22], colorectal [23], or lung [24] have already shown that a tumor-agnostic approach with a biomarker panel assay containing the most frequent mutations in these tumors is a feasible strategy for monitoring patients.

Here, in an independent series of MIBC patients, we evaluated the two most frequent somatic mutations found in our previous cohort (69% *TERT* c.1-124C>T and 42% *ATM* c.1236-2A>T) [13]. Our results show that around 40–50% (depending on the follow-up time point) of MIBC patients harbored the *TERT* mutation in their plasma cfDNA samples. Similarly, several studies have described a high percentage of cancer patients, including BC patients, with the *TERT* c.1-124C>T mutation [14,25,26,27,28,29]. Additionally, we found that the mean MAF of *TERT* c.1-124C>T was significantly higher in progressive than non-progressive MIBC patients, as well as in those patients who died due to BC, both before RC and 12 months later, confirming our previous results [13]. These data suggest that radical surgery could reduce the MAF of *TERT* c.1-124C>T in the bloodstream and that an increase in the MAF of this mutation 12 months after RC would indicate the presence of disease relapse. Furthermore, in accordance with other studies analyzing *TERT* mutations in cancer patients [25,30,31,32,33,34], we found that MIBC patients harboring *TERT* c.1-124C>T had a worse clinical outcome than those without the mutation, emphasizing the aggressive role of the *TERT* mutation in MIBC.

The present study also demonstrates that MIBC patients with a positive ctDNA status at baseline and 4 and 12 months later are at high risk of disease progression, confirming our previous ctDNA testing results using a tumor-informed approach [13]. Similarly, Shohdy et al. [19], using a tumor-agnostic 73-gene assay approach (Guardant360^®^ NGS commercial assay), analyzed the ctDNA status of 53 MIBC patients, demonstrating that those patients with a positive ctDNA status at baseline had shorter overall survival.

Finally, we analyzed the dynamic ctDNA status and demonstrated that, after RC, patients with a positive ctDNA status had the worst prognosis, while patients who maintained a negative status had the best clinical outcomes. This change from negative to positive ctDNA status likely indicates a false-negative result at baseline; the use of a larger biomarker panel could settle this discrepancy. On the other hand, ctDNA clearance after surgery was strongly associated with favorable outcomes, according to our previous study and those of other authors [13,19,20,35]. These results further support the usefulness of longitudinal ctDNA analysis to monitor MIBC patients undergoing RC.

The strength of this work lies in the fact that it is the first to demonstrate that a tumor-agnostic approach composed of BC-specific gene assays is a feasible strategy for monitoring MIBC patients after RC. The results obtained herein are comparable with our previous results with a tumor-informed approach. The main advantage of tumor-agnostic ctDNA testing is its easy translation to the clinical setting, as it allows for monitoring of MIBC patients regardless of their patient-specific genomic variants and, therefore, avoids individual tumor analysis and patient-specific ctDNA assays. In addition, the technical approach used in this study, ddPCR, is affordable and usually already present in routine diagnostic laboratories, which would further promote the translation of ctDNA testing to the clinical setting.

We must acknowledge some study limitations. Firstly, we used only two mutations to detect ctDNA in plasma samples; some patients might not harbor these specific mutations. Moreover, the molecular features of MIBC patients can be modified during their follow-up due to, for instance, resistance mechanisms in patients receiving adjuvant treatments. In addition, there may be low levels of ctDNA in plasma at the time of RC due to the non-shedding of the tumor, resulting in a false-negative result at this time point. Finally, although 162 plasma samples were analyzed in this study, our cohort is limited. Therefore, a final validation of our results in a larger independent series is necessary to define the true role of serial ctDNA monitoring in MIBC patients after RC using a tumor-agnostic approach.

## 4. Materials and Methods

### 4.1. Patients and Samples

A total of 42 consecutive MIBC patients [median age (range) 67 years (50–80); 39 males, 3 females] who underwent RC and extended lymphadenectomy at our center (Hospital Clinic of Barcelona, Barcelona, Spain) between 2019 and 2022 were prospectively included. The exclusion criterion was the presence of another active neoplasm. Follow-up data were available for all patients. Tumor dissemination was controlled postoperatively via computed tomography scans at three-month intervals for the first year, six-month intervals for the next two years, and annually thereafter. Tumors were considered as progressing when relapses or distant metastases developed during follow-up. PFS and CSS were measured from the date of RC to the event. A final follow-up date was registered in cases without events.

One 10 mL EDTA tube of peripheral blood was collected before RC and 1, 4, and 12 months after surgery. Blood samples were stored at 4 °C until processed within the following 6 h.

All methods were carried out in accordance with relevant guidelines and regulations. All patients provided written informed consent (HCB/2013/8753) before being enrolled in this study. The study methodologies conformed to the standards set by the Declaration of Helsinki and were approved by the Clinical Research Ethics Committee of the Hospital Clinic of Barcelona (HCB/2018/0026).

### 4.2. Blood Sample Procedures

Blood samples were centrifuged at 3500 rpm for 15 min at 4 °C to separate plasma, followed by plasma centrifugation at 16,000× *g* for 10 min at 4 °C to remove any remaining cells. The plasma samples were stored at −80 °C until cell-free DNA (cfDNA) extraction.

cfDNA was extracted from 2 to 5 mL of plasma (depending on availability) using the QIAamp Circulating Nucleic Acid kit (Qiagen, Hilden, Germany), according to the manufacturer’s instructions, and quantified using the Quant-iT PicoGreen dsDNA kit (Thermo Fisher Scientific, Waltham, MA, USA), in accordance with the manufacturer’s instructions.

### 4.3. Droplet Digital PCR

The determination of ctDNA status in each patient was performed by detecting two specific tumor mutations in their cfDNA samples via droplet digital PCR (ddPCR). These two most frequent somatic mutations identified in our previous study (*TERT* c.1-124C>T and *ATM* c.1236-2A>T) [13] were analyzed in a total of 162 plasma cfDNA samples from 42 MIBC patients before RC and at three time points during the first year of follow-up (1, 4, and 12 months) to determine ctDNA status at each follow-up time point. The ddPCR experiments were performed using the QX200 Droplet Digital OCR system (Bio-Rad Laboratories, Hercules, CA, USA), according to the manufacturer’s instructions. The assays used to determine both mutant and wild-type amplicons for each specific mutation were designed and tested as reported in our previous study [13]. Further details about the ddPCR experiments can be checked in our previous study [13].

Data analysis was carried out using QuantaSoft Analysis Pro Software, v1.0 (Bio-Rad Laboratories, Hercules, CA, USA). Original figures pertaining to ddPCR analyzing both *TERT* and *ATM* mutations are shown in Figure S4.

### 4.4. Statistical Analysis

Differences in the MAF of both *TERT* and *ATM* mutations at different follow-up time points were analyzed using the Mann–Whitney U-test for independent samples and the Friedman test for repeated measures. *TERT* c.1-124C>T and *ATM* c.1236-2A>T mutations were considered positive if the MAF value for each mutation was higher than the cut-off established in our previous study (0.2 for *TERT* and 0.06 for *ATM*) [13]. Additionally, a ctDNA sample was considered positive if at least one of the two mutations had an MAF value higher than these cut-off values. Using this definition, 31% (50/162) of all plasma samples were ctDNA-positive. Kaplan–Meier curves were generated and compared using log-rank tests. A Cox regression analysis was performed on ctDNA status at different follow-up time points and clinicopathological variables (pathological stage, lymph node status, and neo/adjuvant treatments) to examine their influence on PFS and CSS. Additionally, Cox regression analysis was performed on ctDNA status dynamic changes between RC and four months later to examine their influence on PFS. Statistical significance was established at a *p*-value of 0.05. All analyses were carried out with the SPSS software package (IMB SPSS Statistics 25).

## 5. Conclusions

Here, we show that serial ctDNA analysis using a tumor-agnostic approach is a powerful tool for monitoring MIBC patients after RC. We found that a positive ctDNA status is a prognostic biomarker of tumor progression at baseline and at 4 and 12 months after RC. Moreover, patients harboring the *TERT* c.1-124C>T mutation before RC and 12 months later are at high risk of recurrence. Finally, we show that dynamic ctDNA status analysis has prognostic implications for MIBC patients after RC. The implementation of tumor-agnostic ctDNA testing in the clinical setting could improve disease management and patients’ clinical outcomes.

## Figures and Tables

**Table 1:** Clinicopathological features of MIBC patients enrolled in this study.

	TOTAL MIBC	Progressive MIBC	Non-Progressive MIBC
	N (%)	N (%)	N (%)
	(N = 42)	(N = 10)	(N = 32)
Gender			
Male	39 (93)	9 (90)	30 (94)
Female	3 (7)	1 (10)	2 (6)
Median Age (yr)	67	69	66
Pathological Stage			
<pT2	19 (45)	2 (20)	17 (53)
pT2	11 (26)	2 (20)	9 (28)
pT3	8 (19)	2 (20)	6 (19)
pT4	4 (10)	4 (40)	-
Lymph Nodes (LN)			
LN+	13 (31)	7 (70)	6 (19)
<pT2	2 (5)	1 (10)	1 (3)
pT2	3 (7)	-	3 (9)
pT3	4 (10)	2 (20)	2 (6)
pT4	4 (10)	4 (40)	-
LN-	29 (69)	3 (30)	26 (81)
Neoadjuvant Chemotherapy	15 (38)	3 (30)	12 (38)
<pT2	10 (24)	1 (10)	9 (28)
pT2	2 (5)	1 (10)	1 (3)
pT3	3 (7)	1 (10)	2 (6)
pT4	-	-	-
Adjuvant Chemotherapy	5 (12)	1 (10)	4 (13)
<pT2	-	-	-
pT2	2 (5)	-	2 (6)
pT3	2 (5)	-	2 (6)
pT4	1 (2)	1 (10)	-

Abbreviations: LN; lymph nodes; MIBC; muscle-invasive bladder cancer.

## Data Availability

Data are available upon reasonable request.

## Supplementary Materials

The following supporting information can be downloaded at: https://www.mdpi.com/article/10.3390/ijms242316578/s1.
