# Supplementary material for: Tumor-Agnostic Circulating Tumor DNA Testing for Monitoring Muscle-Invasive Bladder Cancer
Source: Int J Mol Sci. 2023 Nov 21;24(23):16578. doi: 10.3390/ijms242316578 (PMC10706140; doi:10.3390/ijms242316578)

SUPPLEMENTARY TABLES

Table S1. Mean MAF of *TERT* c.1-124C>T and *ATM* c.1236-2A>T in (A) progressive and non-progressive MIBC patients and (B) patients who died due to BC at the different follow-up time points.

A)

	Cystectomy		1 month		4 months		12 months	
	Progressive (N=10)	Non-Progressive (N= 32)	Progressive (N= 10)	Non-Progressive (N= 32)	Progressive (N= 10)	Non-Progressive (N= 32)	Progressive (N= 6)	Non-Progressive (N= 31)
TERT c.-124C>T	0.53* (N= 7)	0.13* (N= 11)	0.23 (N= 3)	0.15 (N= 7)	0.41 (N= 6)	0.13 (N= 14)	2.93* (N= 4)	0.06* (N= 8)
ATM c.1236-2A>T	0.17 (N= 3)	0.05 (N= 5)	0.00 (N= 0)	0.04 (N= 6)	0.04 (N= 1)	0.02 (N= 3)	0.15 (N= 3)	0.00 (N= 0)

*There were significant differences ($p<0.05$) between progressive and non-progressive patients.

B)

	Cystectomy		1 month		4 months		12 months	
	CSD Patients (N= 6)	Remaining Patients (N= 36)	CSD Patients (N= 6)	Remaining Patients (N= 36)	CSD Patients (N= 6)	Remaining Patients (N= 36)	CSD Patients (N= 2)	Remaining Patients (N= 35)
TERT c.-124C>T	0.65* (N= 5)	0.15* (N= 13)	0.33 (N= 2)	0.15 (N= 8)	0.58 (N= 4)	0.13 (N= 16)	8.46* (N= 2)	0.08* (N= 10)
ATM c.1236-2A>T	0.26 (N= 2)	0.05 (N= 6)	0.00 (N= 0)	0.03 (N= 6)	0.00 (N= 0)	0.03 (N= 4)	0.00 (N= 0)	0.02 (N= 3)

Remaining patients: patients alive or dead due to non-tumor causes.

Abbreviation: CSD, cancer-specific death

*There were significant differences ($p<0.05$) between CSD and the remaining patients.

SUPPLEMENTARY FIGURES

Figure S1. Percentage of patients presenting *TERT* or *ATM* mutations in cfDNA samples at different follow-up time points.

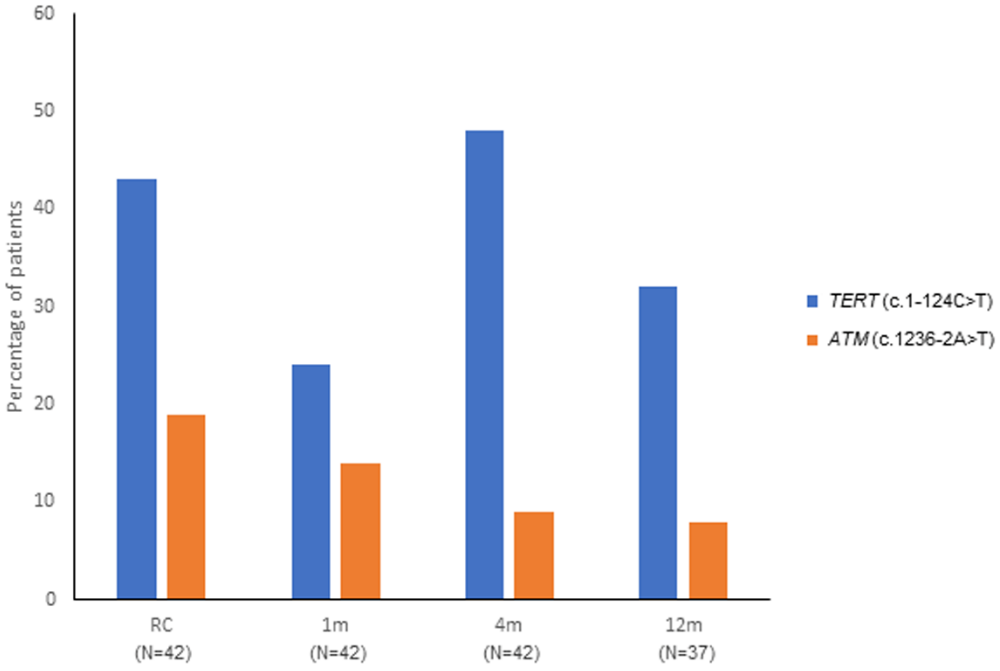

Abbreviations: RC; radical cystectomy, 1m; 1 month after RC, 4m; 4 months after RC, 12m; 12 months after RC.

Figure S2. Mean MAF of *TERT* c.1-124C>T in MIBC patients according to (A) tumor progression and (B) death occurrence at each follow-up time point.

A)

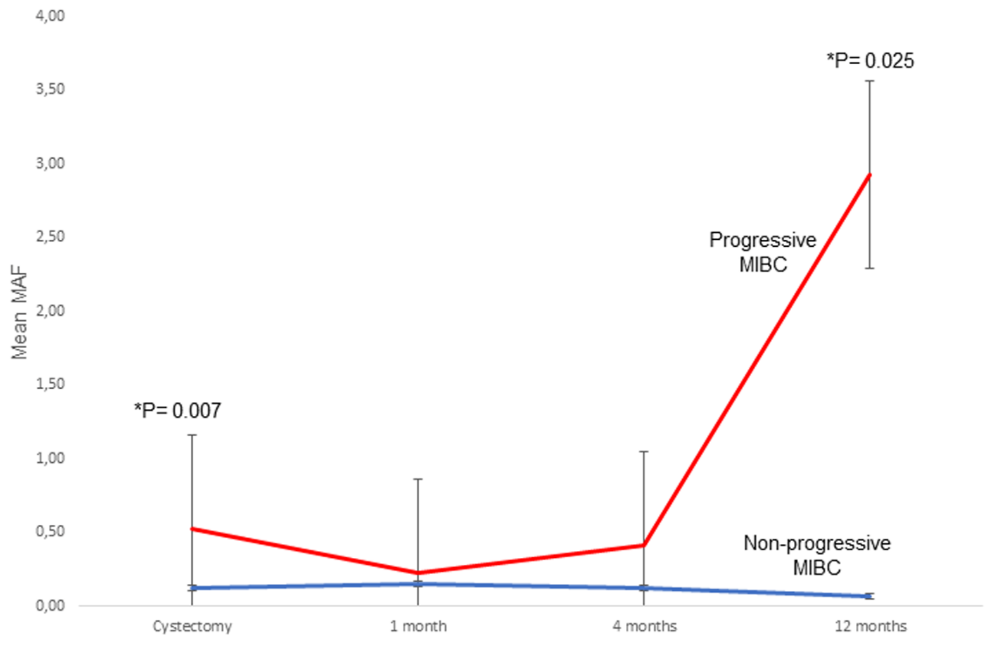

B)

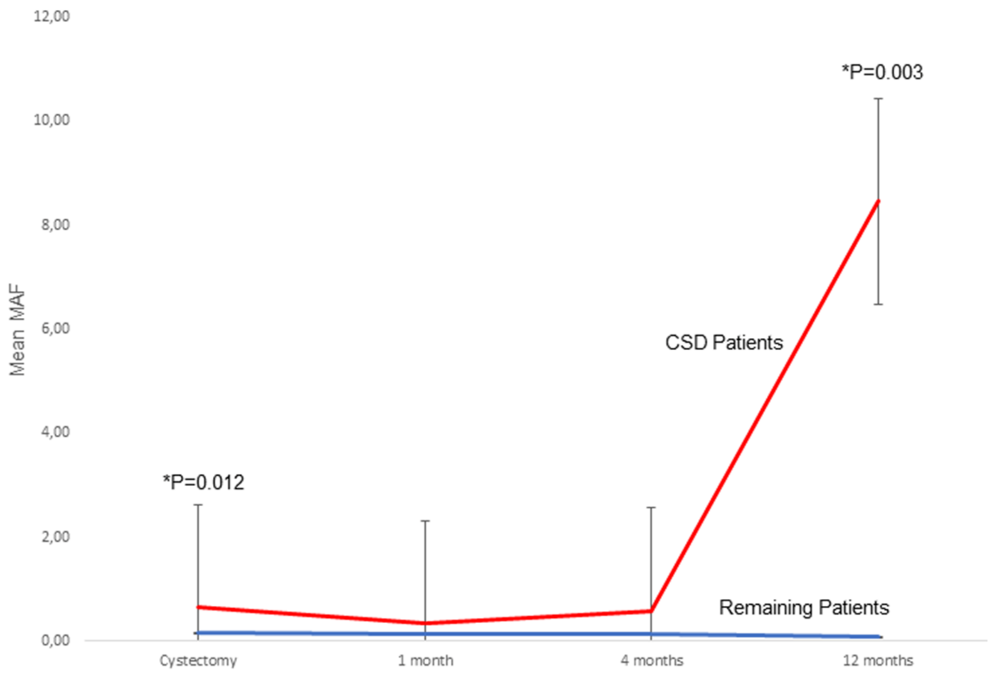

Remaining patients: patients alive or dead due to non-tumor causes.

Abbreviation: CSD, cancer-specific death; MAF, mutation allele fraction.

Figure S3. Prognostic value of the *TERT* c.1-124C>T mutation at different time points. Kaplan-Meier survival analysis shows the probability of tumor progression in MIBC patients stratified by *TERT* mutation status (positive or negative) (A) at baseline and (B) four months after RC. It also shows the probability of cancer-specific survival in MIBC patients stratified by *TERT* mutation status (positive or negative) (C) at baseline and (D) four months after RC. The MAF of *TERT* c.1-124C>T above or below the cut-off is considered positive or negative, respectively.

A)

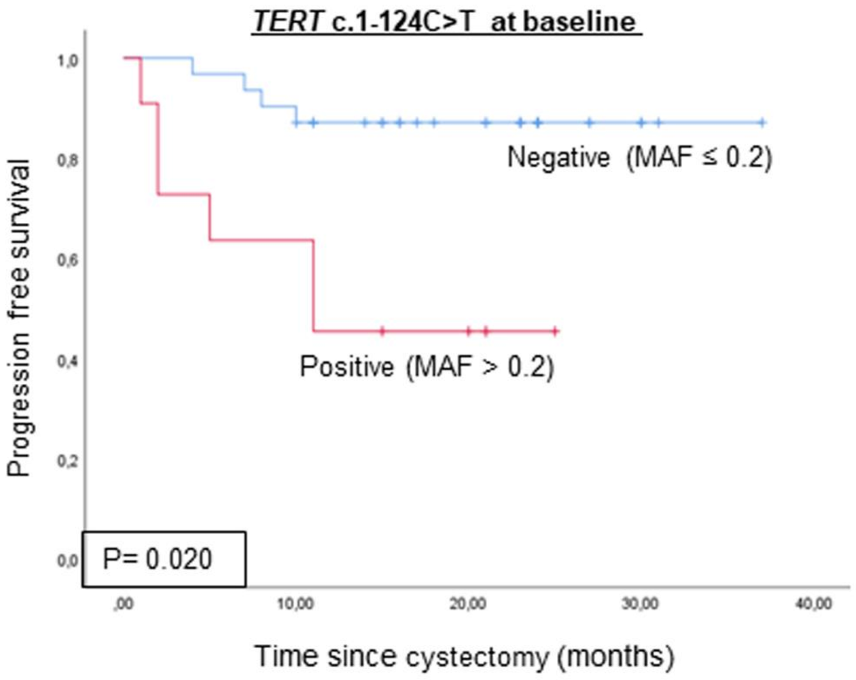

B)

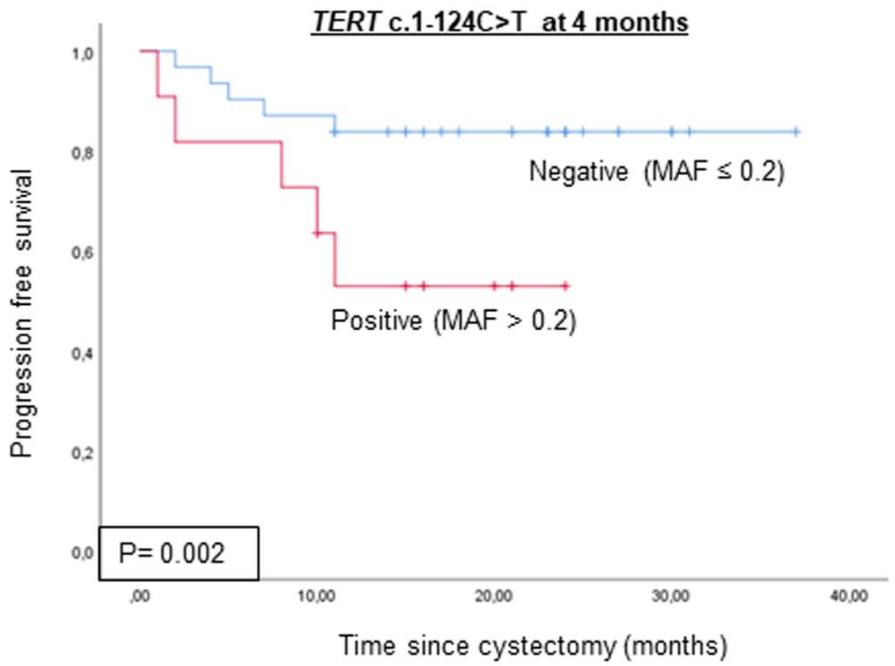
TERT c.1-124C>T at 4 months
Progression free survival
1,0
0,8
0,6
0,4
0,2
0,0
Negative (MAF ≤ 0.2)
Positive (MAF > 0.2)
P= 0.002
,00
10,00
20,00
30,00
40,00
Time since cystectomy (months)

C)

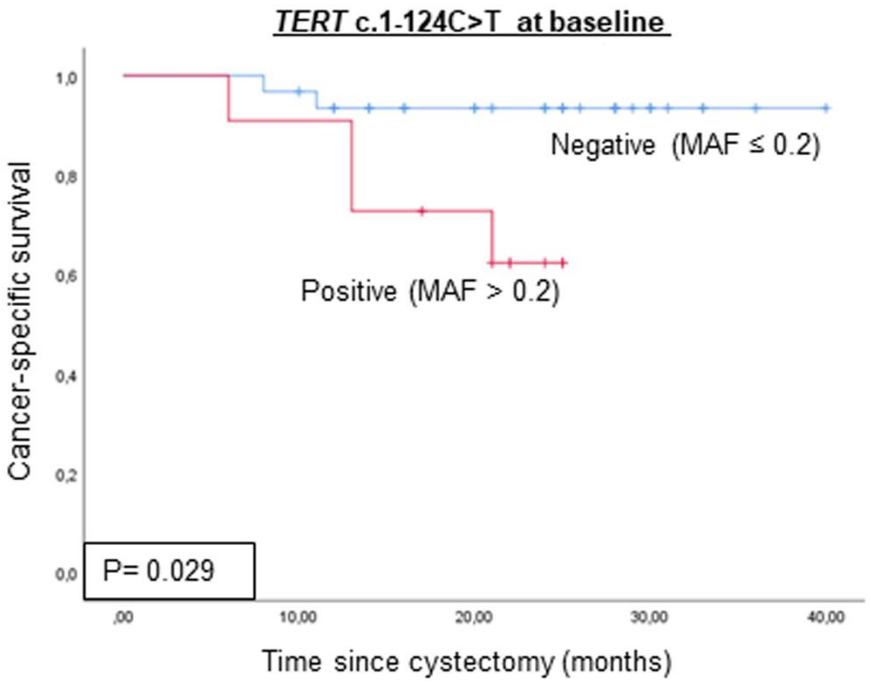
TERT c.1-124C>T at baseline
Cancer-specific survival
1,0
0,8
0,6
0,4
0,2
0,0
Negative (MAF ≤ 0.2)
Positive (MAF > 0.2)
P= 0.029
,00
10,00
20,00
30,00
40,00
Time since cystectomy (months)

D)

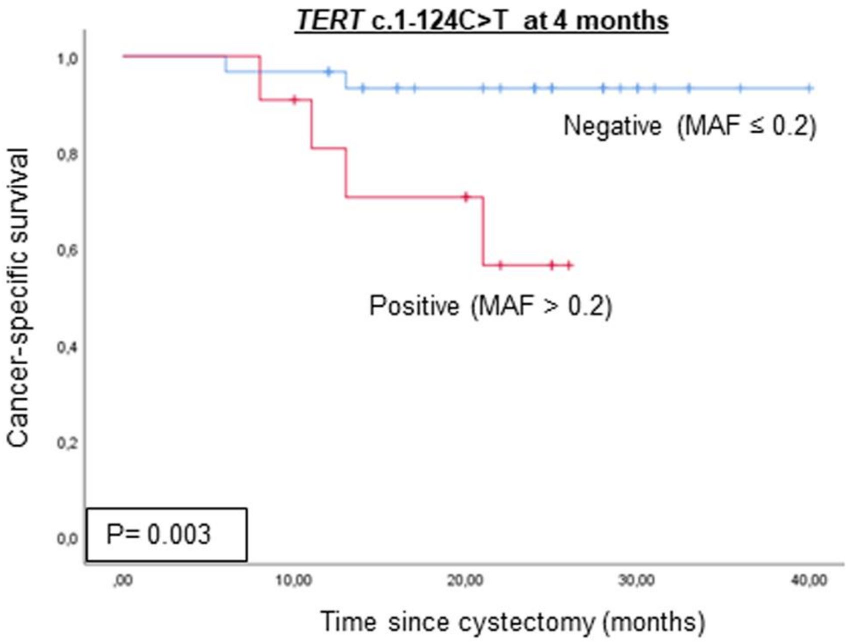

Abbreviations: MAF, mutation allele fraction.

Figure S4. Representative two-dimensional (2D) scatter spot plots of ddPCR results analyzed through the QuantaSoft Analysis Pro Software (Bio-Rad). The 2D amplitude plots show an example of both mutated and non-mutated samples for (A) the *TERT* c.1-124C>T assay and (B) the *ATM* c.1236-2A>T assay. The y-axis shows the fluorescence amplitude of the FAM probe (Channel 1), designed to hybridize only with the wild-type allele (blue). The HEX probe, which hybridizes only with the mutant allele (green), is plotted on the x-axis (Channel 2). Double-positive droplets carrying both mutant and wild-type alleles are represented in orange, while double-negative droplets (no amplification) are shown in grey.

A) *TERT* c.1-124C>T

Mutated sample

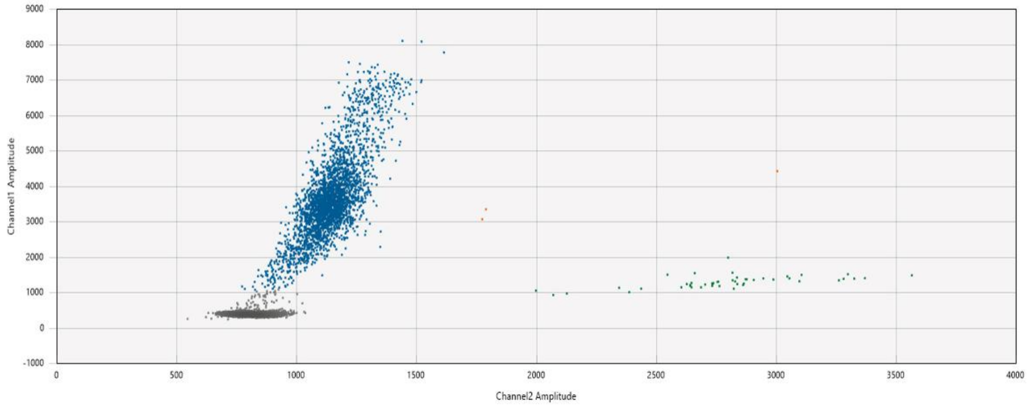

Non-mutated sample

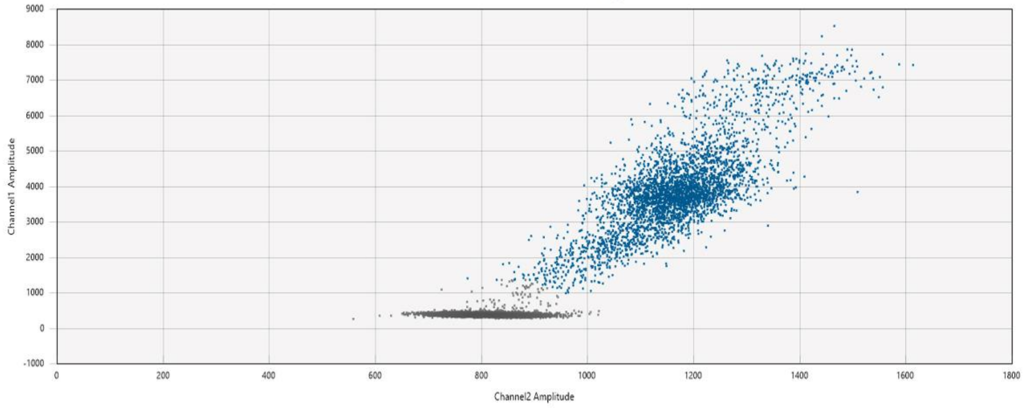

B) *ATM* c.1236-2A>T

Mutated sample

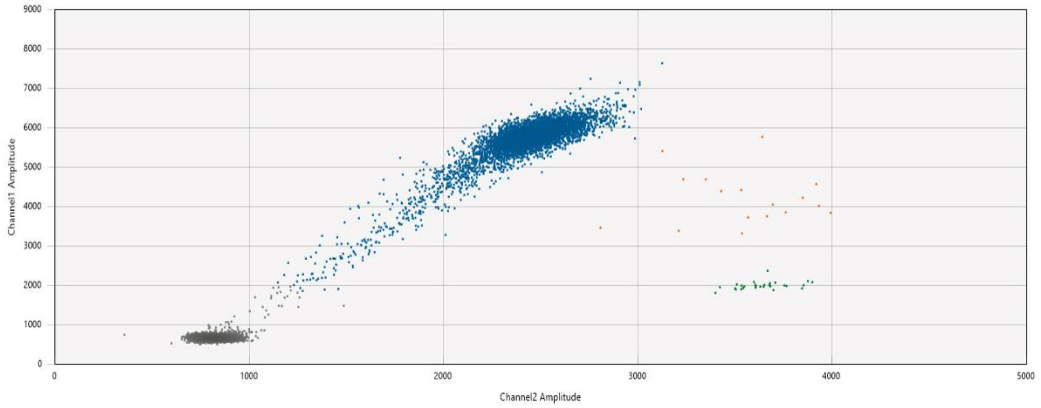

Non-mutated sample

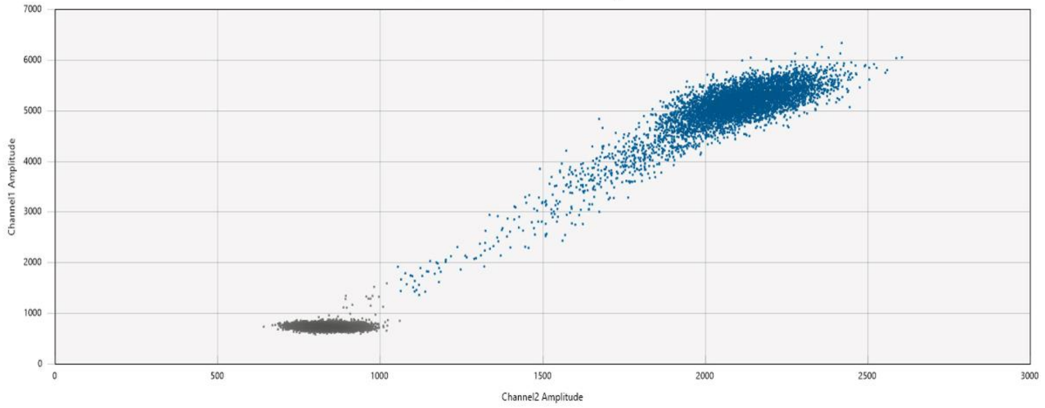

Supplement: Supplementary file 1 [file ijms-24-16578-s001.zip › ijms-2730137-supplementary.pdf]
